# Chondro-Osseous Metaplasia in Ependymoma: A Rare Histopathological Finding

**DOI:** 10.1155/2020/1528698

**Published:** 2020-05-05

**Authors:** Ali Alkhaibary, Fahd AlSufiani, Ali H. Alassiri, Makki Almuntashri, Salma Tarik Al Qutub

**Affiliations:** ^1^College of Medicine, King Saud bin Abdulaziz University for Health Sciences, Riyadh, Saudi Arabia; ^2^King Abdullah International Medical Research Center, Riyadh, Saudi Arabia; ^3^Ministry of National Guard-Health Affairs, Riyadh, Saudi Arabia; ^4^Department of Pathology and Laboratory Medicine, King Abdulaziz Medical City, Riyadh, Saudi Arabia; ^5^Medical Imaging Department, King Abdulaziz Medical City, Riyadh, Saudi Arabia; ^6^Diagnostic Neuroradiology Department, King Faisal Specialist Hospital, Dr. Fakeeh Hospital, Jeddah, Saudi Arabia

## Abstract

Ependymoma is a circumscribed glioma composed of uniform glial cells with bland nuclei in a fibrillary matrix. It is characterized by the presence of perivascular pseudorosettes. Unusual histopathological findings have rarely been reported in ependymomas, 0.5% of all diagnosed cases. Such unusual and exceedingly rare histological findings include osseous or chondroid metaplasia. To the best of our knowledge, only 15 cases of osseocartilaginous ependymomas have been reported in English literature. We report a 3-year-old boy who presented with ataxia, vomiting, and headache for three months. Radiological imaging revealed a posterior fossa lesion. Histopathological examination of the lesion confirmed a posterior fossa ependymoma with chondro-osseous metaplasia. The present case outlines the clinical presentation, histopathological findings, and outcome of chondro-osseous metaplasia in ependymomas. To date, the etiology of chondro-osseous metaplasia in ependymomas remains uncertain. Further research exploring such phenomenon is of paramount importance to explain how these tumors develop.

## 1. Introduction

Ependymoma is a noninfiltrative glioma usually arising near the ventricular system or central canal. Half of all ependymomas, particularly those in the posterior fossa, occur in children, making them the second most frequent solid brain tumor in the pediatric population after medulloblastoma [1]. Ependymomas represent 5.7% of all diagnosed CNS tumors in children aged 1-14 years with an estimated annual incidence rate of 0.30 per 100,000 people [2]. The incidence of ependymoma is higher in males [1]. Ependymomas tend to have a variable clinical outcome, dependent upon the extent of surgical resection, adjuvant radiotherapy, and the molecular classification [3].

Traditionally, according to the WHO grading scheme, ependymomas are divided into three types. These include myxopapillary ependymoma (WHO grade I), conventional ependymoma (WHO grade II), and anaplastic ependymoma (WHO grade III). Unusual histopathological patterns such as pigmented (melanotic) ependymomas, giant-cell ependymomas, ependymomas with extensive tumor cell vacuolization, and chondro-osseous ependymomas have been reported in the literature. These rare patterns are noted in only 0.5% of all diagnosed ependymomas [4].

The presence of bony or cartilaginous differentiation in gliomas is extremely rare and has been recognized in fourth-ventricular ependymomas and midline astrocytomas [5–9]. A review of the English literature reveals 15 cases of chondro-osseous ependymomas. We report a case of ependymoma with chondro-osseous metaplasia by outlining the clinical presentation, histopathological features, and outcome.

## 2. Case Description

**Figure 1:**
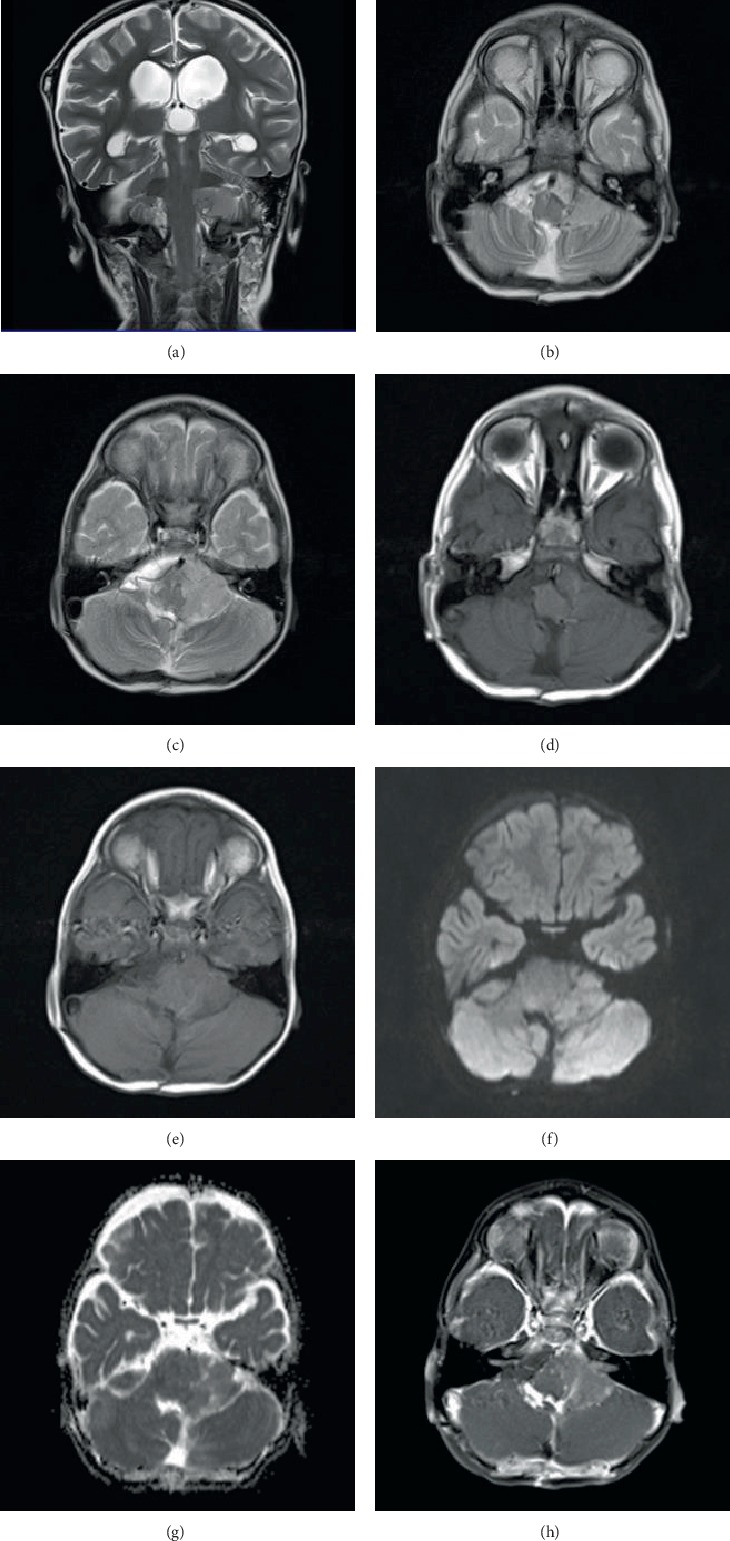
(a) Coronal T2 WI. (b, c) Axial T2 WI. (d, e) Axial T1 WI. (f, g) Axial DWI and ADC map. (h) Axial T1 postcontrast. (a–h) A large soft tissue mass is noted at the left cerebellopontine angle demonstrating an intermediate hyperintense signal on T2WI and hypointense signal on T1WI with mild restriction on DWI and faint homogenous enhancement after contrast. (a) The mass is causing hydrocephalus which has resolved with bilateral small subdural collections, related to the reduced intracranial pressure, following surgery.

A 3-year-old boy presented with a 3-month history of ataxia, vomiting, and headache, quickly followed by signs of increased intracranial pressure (ICP). Investigations and radiological imaging in private hospitals identified a posterior fossa space-occupying lesion (Figure 1). Consequently, ventricular shunting and a subtotal resection (STR) were performed which were complicated by a moderate posterior fossa syndrome.

### 2.1. Histopathological Findings

**Figure 2:**
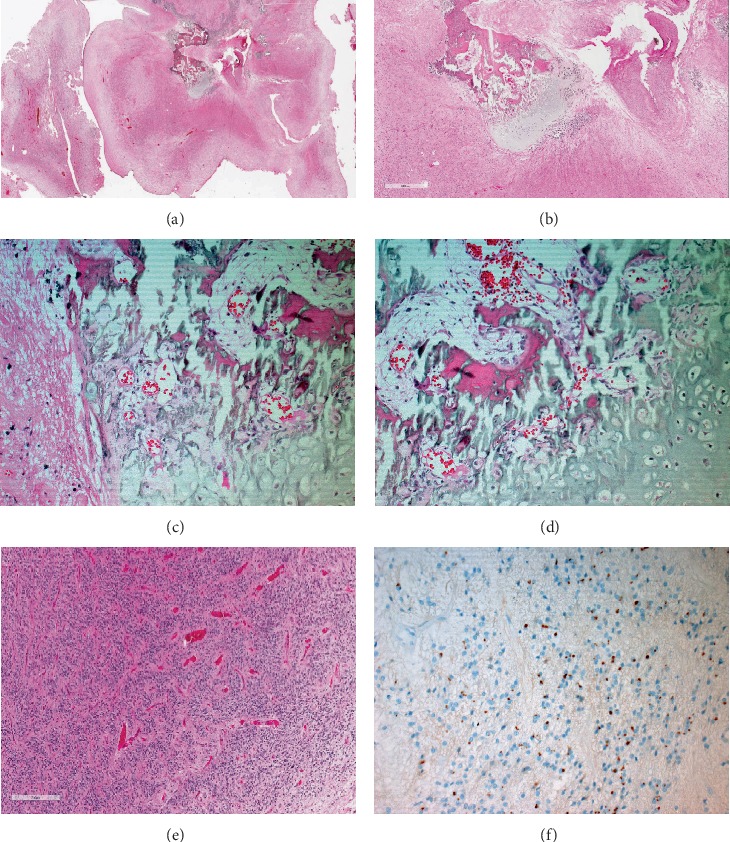
(a) A panoramic view of chondro-osseous ependymoma. (b) Very low magnification (20×) of the hematoxylin and eosin-stained section depicting chondro-osseous metaplasia within the substance of conventional ependymoma (areas of anaplasia are not shown). (c, d) At slightly higher magnification (200× and 400×), the organized hyaline cartilage formation and ossification is appreciated. (e) Another low-power field (20×) exhibiting the classic ependymal differentiation in the form of pseudorosettes. (f) Dot-like staining by EMA immunostain.

The histopathological examination of the posterior fossa tumor showed mostly a moderately cellular glial tumor with perivascular pseudorosettes (Figure 2). There are foci of hypercellularity, pleomorphism, and increased mitotic activity (up to 10 mitoses per 10 HPF). The tumor cells are immunopositive for GFAP. EMA immunostain showed a perinuclear dot-like pattern (Figure 2(f)). The histopathology and the immunoprofile are classical for ependymoma with a focus of anaplasia (WHO III). In addition, there was a focus of chondro-osseous metaplasia within the well-differentiated part of the tumor. There is a rim of dystrophic calcification adjacent to this metaplasia as well.

### 2.2. Outcome and Follow-Up

After four months of the STR, the patient underwent gross total resection to remove the residual tumor. Afterwards, the patient completed radiation therapy. Routine follow-up after two years revealed that the boy's speech was coherent with no oropharyngeal deficits. The VP shunt was compressible, filling promptly on 2.0 pressure, with no signs of malfunction. He was able to ambulate independently with minimal residual gait ataxia noted when running. The patient was otherwise grossly intact.

Subsequent radiological imaging revealed no evidence of residual tumor, recurrence, or drop metastasis. The cerebrospinal fluid and cytopathological analyses were negative for malignant cells. The patient is currently followed up in the pediatric neurosurgery clinic.

## 3. Discussion

Chondro-osseous metaplasia in ependymomas is exceedingly rare. An extensive review of the literature revealed 15 prior cases of chondro-osseous ependymomas (Table 1). We report an additional case encountered at our institution. Most of the reported cases (*N* = 11; 68.7%) were diagnosed in the pediatric age group (defined as less than or equal to 16 years). The youngest patient who was diagnosed with ependymoma with chondroid metaplasia was one year old (age range: 1–61 years).

Of the fifteen reported cases, six depicted both chondroid and osseous metaplasia with no gender predilection. This makes the present case the seventh of its kind. The majority of the tumors were localized in the posterior fossa (*N* = 14; 87.5%) with the remaining tumors in the frontal lobe, temporo-occipital lobes, and cerebellopontine angle cistern.

Histologically, seven patients (43.7%) had conventional ependymoma (WHO II), six patients (40%) had anaplastic ependymoma (WHO III), one patient had subependymoma/ependymoma, and one had myxopapillary ependymoma. As far as the outcome is concerned, six patients (37.5%) passed away within three years of the diagnosis, one patient (6.2%) was lost to follow-up, and two patients (13.3%) had an uneventful outcome. The boy in the present case had no recurrence of the tumor after two years of the diagnosis.

According to Wang et al., the presence of chondro-osseous metaplasia in ependymomas is associated with a dismal prognosis despite aggressive therapy, concluding that such tumors may behave aggressively [9]. Boukas and his group reported the first case of ependymoma in which cartilaginous metaplasia has replaced the entire architecture of the tumor. They considered the presence of chondro-osseous metaplasia in ependymomas an obstacle to achieve gross total resection [7].

Several mechanisms have been postulated to explain chondro-osseous metaplasia in ependymoma [9]. Two of the most accepted theories state that such a phenomenon in gliomas may arise due to the metaplastic transformation of either the neoplastic glial cells or the mesenchymal tissue component of the tumor [16]. Güzey et al. reported a case of ependymoma with cartilage formation. They concluded that the cartilage formation might have developed from a transformation of the neuroepithelial cells to mesenchymal cells, as the cartilaginous tissue lacked a fibrous capsule and immunoreacted positively to glial fibrillary acidic protein (GFAP) antibody [6].

Gessi et al. identified four patients with cartilaginous ependymomas in his case series. They were not able to establish any relationship between the tumor grade and the presence of chondro-osseous metaplasia. In his case series, there were no high-grade features. They postulated that chondro-osseous metaplasia could have resulted from a simple transformation of mesenchymal stromal tissue within the tumor [4].

The capacity of neoplastic cells to produce cartilage in gliomas may be related to their ability to secrete and synthesize basement membrane material which, upon condensation, may become a chondroid substance [14]. However, considering the rarity of this histopathological feature, no single mechanism could explain the presence of osseocartilaginous metaplasia in ependymomas [6].

## 4. Conclusion

We report a case of posterior fossa ependymoma with chondro-osseous metaplasia. Although rare, such metaplastic changes can be identified in ependymomas and the prognostic significance is uncertain.

## Figures and Tables

**Table 1:** Summary of the reported cases of chondro-osseous ependymoma in the literature.

No.	1^st^ Author (year)	Age^∗^/sex	Localization	Histology	WHO grade	Metaplasia	Outcome
1	Ghosal N [10], 2010	16/M	4^th^ ventricle	Ependymoma	II	Chondroid	Lost to follow-up
2	Coli A [11], 2014	5/M	4^th^ ventricle	Anaplastic	III	Chondroid	Death at 36 months
3	Boukas A [7], 2013	5/M	4^th^ ventricle	Anaplastic	III	Chondroid	Death at 3 months
4	Wang X [9], 2012	5/M	4^th^ ventricle	Ependymoma	II	Chondro-osseous	Death at 18 months
5	Jain A [8], 2009	21/M	4^th^ ventricle	Ependymoma	II	Chondroid	Recurrence at 5 years
6	Mridha AR [12], 2007	9/M	Lt. CP angle cistern	Anaplastic	III	Chondro-osseous	NA
7	Siqueira EB [13], 1966	10/F	4^th^ ventricle	Ependymoma	II	Chondroid	Death
8	Kepes JJ [14], 1984	7/F	4^th^ ventricle	Ependymoma	II	Chondroid	Death at 5^th^ day post-operation
9	Güzey FK [6], 2005	56/F	Rt. Temporo-occipital lobe	Ependymoma	II	Chondroid	No complaints at 15 months
10	Bannykh S [15], 2007	61/M	Lt. Frontal lobe	Anaplastic	III	Chondro-osseous	Death at 6 months
11	Chakraborti S [16], 2012	50/F	4^th^ ventricle	Myxopapillary	I	Chondroid	No recurrence at 3 years
12	Gessi M [4], 2011	1/F	4^th^ ventricle	Anaplastic	III	Chondroid	NA
13	Gessi M [4], 2011	2/F	4^th^ ventricle	Anaplastic	III	Chondro-osseous	NA
14	Gessi M [4], 2011	3/F	4^th^ ventricle	Ependymoma	II	Chondro-osseous	NA
15	Gessi M [4], 2011	53/F	4^th^ ventricle	Subependymoma/Ependymoma	I	Chondro-osseous	NA
16	Present Case, 2020	3/M	4^th^ ventricle	Anaplastic	III	Chondro-osseous	No recurrence at 2 years

^∗^Age is expressed in years. CP: Cerebellopontine; M: male; F: female; Rt: right; Lt: left; WHO: World Health Organization; NA: not available.
